# Positron emission tomography in the management of lung cancer

**DOI:** 10.4103/1817-1737.32235

**Published:** 2007

**Authors:** Vahid Reza Dabbagh Kakhki

**Affiliations:** *Assistant Professor, Department of Nuclear Medicine, Imam Reza Hospital, Mashhad University of Medical Sciences, Mashhad, Iran*

**Keywords:** Lung cancer, positron emission tomography (PET), PET/computed tomography, tumor staging, therapy response

## Abstract

^18^ F-fluorodeoxyglucose positron emission tomography (FDG-PET) is a useful technique to characterize the solitary pulmonary nodule, diagnose primary lung cancer, carry out mediastinal and extrathoracic staging, plan radiotherapy, therapeutic response assessment and detect recurrence. PET may help to determine the ideal site for tissue diagnosis as well as predict prognosis. Combined PET and computed tomography (PET / CT) has the best of both worlds of metabolic and anatomic imaging and may provide optimal disease assessment.

Imaging plays a vital role in the diagnosis, staging, therapeutic assessment and follow-up of patients with lung cancer. In the past decade, FDG-PET has become a major adjunct to structural imaging techniques because it adds a biological dimension by the study of glucose metabolism in tissues. The development of integrated positron emission tomography and computed tomography (PET/CT) scanning has made it possible to acquire both morphological and functional information of the entire body in a single examination.[1] This article provides a brief history of PET and PET / CT imaging, a review of the current PET literature pertaining to lung cancer and gives specific recommendations for its use.

## PET and PET/CT

Whole-body PET scanners provide the unique ability to quantitate metabolic processes *in vivo*. Tumors exhibit an accelerated glycolysis allowing ^18^F-fluorodeoxyglucose (FDG, an analog of glucose) to be trapped in tissues with an elevated metabolic rate compared with normal tissues.[2,3] Although PET images show functional information, they provide limited anatomical data, which in regions such as the head and neck, mediastinum and the pelvic cavity, is a significant drawback. The exact localization of lesions may also be difficult in some cases on the basis of PET images alone.[3–5] In the last 2 years, PET imaging in oncology has been migrating from the use of dedicated PET scanners to the use of PET / CT tomographs.[4]

Researchers realized early on that it would be ideal if the morphological information from CT and the functional information from PET were combined.[6,7] Software registration techniques and providing fusion images for the separate modalities provided more accurate localization than that provided by visual coregistration. However, erroneous registration resulted in several problems. Especially in the fusion of body images, deformable organs caused various errors. Time differences in imaging created other problems. For example, the contours of the abdomen are dependent on the pallet design; also, gastrointestinal organs move over time. This made accurate registration extremely difficult.

PET/CT was designed to provide the solution to these shortcomings.[7] In a PET/CT scanner, the PET and CT tomographs are housed in a single gantry with a single patient bed and workstation. PET/CT scanners can also be used either as a dedicated PET scanner or as a dedicated CT scanner. Upon reconstruction, both the PET images and the CT images are displayed side by side and overlaid (fused).[5] Use of the CT scan reduces the total PET acquisition time as well as enables improved accuracy and precision attenuation correction of the emission images. In addition, the CT scan gives a more precise localization and interpretation of the hypermetabolic lesions, thanks to the availability of anatomical landmarks.[3,5]

PET/CT solves the problems caused by body contour deformability and time difference, but does not routinely correct for breathing artifacts or inadvertent positioning changes between the two scans. PET/CT has been shown to reduce the false-positive interpretation of physiological uptake such as brown fat, muscle and colon uptake.[7–11] Multiple clinical trials revealed the superiority of a PET/CT image over either modality alone.[7] Clinical FDG studies are generally analyzed using qualitative (visual) and semi-quantitative data. A standardized uptake value (SUV) is a semi-quantitative index of glucose utilization that is obtained by normalizing the accumulation in the abnormal lesion to the injected dose and patient body weight. So SUV is calculated using the following formula:

SUV = mean lesion activity/[injected dose/body weight(g)]. Perhaps calculation of SUV_max_ using the pixel with greatest amount of radioactivity in the image of lesion instead of mean activity of several pixels of the lesion would be more appropriate.[12] Please explain what a pixel is here. Also this statement is confusing-do you mean using the most active pixel for calculation of SUV or just using the most active pixel to determine glucose utilization? If so, it would be better to state this.

## Pulmonary Nodule

The crucial objective in the evaluation of the solitary pulmonary nodule (SPN) is the ability to noninvasively differentiate benign from malignant lesions in the most cost-effective manner before definitive therapy while minimizing patient morbidity and mortality.[13] FDG-PET has proven to be an accurate, noninvasive method for the management of (please substitute jargon for terms found in literature) patients with SPN.[14] The reported sensitivity and specificity have ranged from 89-100% and 77-100% respectively, for the detection of malignant pulmonary nodules.[13]

In addition to the importance of visual analysis of the PET images,[15,16] semi-quantitative analysis using a threshold SUV of > 2.5 for the diagnosis of malignancy in a pulmonary nodule has often been suggested, despite the fact that quite a few lesions with SUV < 2.5 are malignant.[15,17,18] False-positive results are seen in active granulomatous disease (tuberculoma and histoplasmosis) and certain other inflammatory processes due to increased glycolytic activity within the active macrophages. False-negative results may be seen in tissues with low metabolic activity such as bronchoalveolar carcinoma (BAC), pulmonary carcinoids or when the lesion is < 5-7 mm in diameter.[14]

The negative predictive power of PET is sufficiently high to obviate the necessity of a biopsy.[19] If FDG-PET is negative for lesions ≥ 7 mm in diameter, then the process is most likely benign and may be followed with serial surveillance. If the lesion is < 7 mm in diameter, then malignancy cannot be excluded with a negative PET.[14] When FDG-PET is positive, then diagnostic and definitive treatment may be instituted.[14] As seen from previous studies, most lesions that were found positive by PET were either malignant or required specific active management determined from subsequent histopathological analysis.[19] Another challenging field for PET is its additional value in lung cancer screening studies.

A sensitivity of 90% was seen in one of the first studies in this setting[20] requiring additional PET for nodules ≥ 7 mm. In a more recent study,[21] PET was also added to the screening protocol for nodules ≥ 10 mm or growing nodules ≥ 7 mm. The conclusion was that selected use of PET is useful in screening trials because it may minimize unnecessary invasive procedures for benign lesions. It was correctly concluded that PET can be of help in screening detected nodules but that its role there is more limited than in screening the general population presenting with pulmonary nodules.[22]

## Lung Cancer Staging

Because survival is inversely correlated with the stage of the lung cancer, a meticulous staging procedure is required to determine the required treatment and prognosis.[23] CT is frequently unable to discriminate between malignant enlarged mediastinal lymph nodes and those that are enlarged due to benign reactive hyperplasia.[24] In addition, conventional imaging limited to the thorax and upper abdomen is unable to detect more distant metastatic disease which can occur in 9–11% of all patients with non-small cell lung cancer (NSCLC).[24,25] However, FDG-PET has been shown to have greater sensitivity for the detection of metabolically active malignant disease and can lead to changes in initial staging and treatment plans for lung cancer when used in combination with conventional work-up.[26,27] One retrospective study[28] on 198 patients confirmed that the use of PET has an important impact on stage designation and clinical decision-making. PET upstaged 16.2% and downstaged 6.1% of the patients.

## Primary Tumor

CT is excellent for determining the location and anatomic size of the primary mass and its relationship to surrounding structures.[24] Compared to CT, “isolated” PET offers little additional information in the tumor (T) characterization of lung cancer due to its lack of spatial resolution and the invisibility of all but the grossest anatomical landmarks.[29] However, one exception is its usefulness in distinguishing between tumor and postobstructive atelectasis.[30] In addition, PET can be beneficial in evaluating the cause of pleural effusions. Gupta *et al*[31] quote an accuracy rate of 91% for PET in a study of 35 patients with lung cancer and suspected malignant pleural effusion.

However, FDG-PET imaging is of potential use in assessing the metabolic activity of the primary lung cancer, which reflects cell turnover rate and may indicate the biologic aggressiveness of the cancer.[24,32–34] Several studies have shown that the SUV has prognostic value independent of the conventional clinical tumor, node, metastasis (TNM) staging.[35,36] For example, Higashi *et al*[37] demonstrated that a primary tumor with SUV > 5 was associated with a significant increase in postoperative relapse in early stage lung cancer. Thus, PET imaging in initial T staging by predicting the likelihood of tumor recurrence after treatment, may help in selecting which patients are likely to respond to induction therapy before surgery and which patients should receive adjuvant chemotherapy / radiotherapy.[24,37]

**Figure 1:**
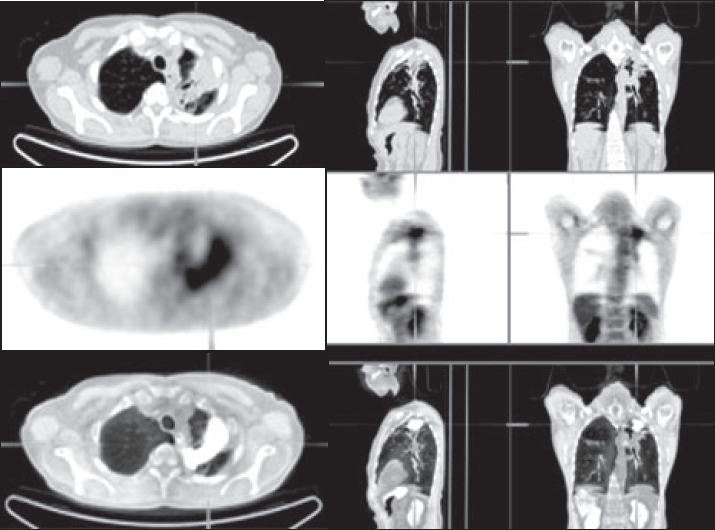
Non-small cell lung cancer. PET/CT images show invading the visceral pleura without chest wall invasion while on the CT alone it would be more difficult to determine the chest wall invasion (T2 N0 M0) (Reprinted with permission of reference).[39]

Integrated PET / CT has been shown to be more useful than dedicated PET imaging in determining the T stage of the primary tumor and in assessing the presence of mediastinal or chest wall invasion[38] [Figure 1]. Halpern *et al*[40] demonstrated an accuracy rate of 97% with PET / CT compared with 67% with PET only. This superiority was attributed entirely to the CT component of the examination. By comparison, another report described accuracy rates for T staging with PET / CT and CT to be 88 and 58%, respectively.[38] The reasons for this surprising finding were not fully explored, but it is worth reiterating that PET can have a role in T staging by distinguishing between tumor and distal atelectasis.[29] It is important to remember that the CT component of PET / CT is acquired without IV contrast in mid-inspiration rather than full inspiration. Therefore, a diagnostic contrast-enhanced CT scan of the chest performed as part of the PET / CT study or independently as a separate scan is still recommended.[24]

## Nodal Involvement

**Figure 2:**
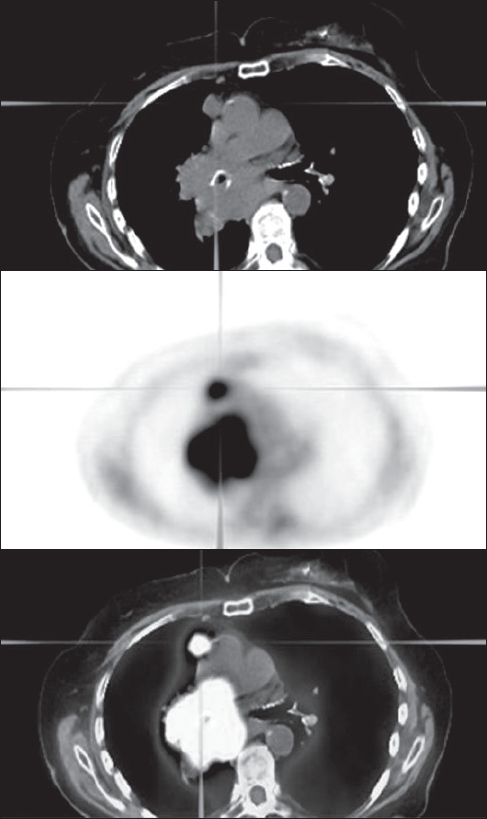
Non-small cell lung cancer. PET/CT images show a mass involving the right hilum with extension into mediastinum without extension to the contralateral mediastinum but a separable focus in the right superior mediastinum (stage IIIA) (Reprinted with permission of reference).[39]

Mediastinal lymph node staging can be divided into imaging and sampling. Analysis of the pooled receiver operating characteristic (ROC) curves indicated that PET was significantly more accurate than CT or MRI in identifying nodal metastasis[41] with a reported accuracy of 81–96%.[24,41] A meta-analysis of different imaging methods for determining nodal stage reported a sensitivity of 79% and a specificity of 91% for the detection of nodal metastases by PET (*vs* 60 and 77% respectively, for CT).[41] Overall, there is 20% improvement in accuracy of PET over CT imaging for mediastinal staging of NSCLC.[14] PET / CT has an even higher diagnostic accuracy than either CT or PET alone[38] with a reported sensitivity of 89% and specificity of 94% and an overall diagnostic accuracy of 93%.[24] A very well-designed prospective study[42] compared CT and PET in the diagnosis of mediastinal lymph node metastases in 132 consecutive patients with potentially resectable NSCLC. Negative predicted probability (NPP) was very high at 98%. The conclusion was that both imaging methods are complementary [Figure 2] and that their common strength is their powerful negative predictive value (NPV). Thus, integrated PET / CT may be more helpful than either of these techniques used alone. False-positive PET results in the mediastinum, which affect selection of treatment, have been reported in the range 13–17% and are mainly due to inflammatory lymph nodes (secondary to pneumonia, postobstructive pneumonitis or chronic granulomatous infection) which may lead to mistaken upstaging of the primary tumor.[24,43,44] Conversely, several reviews have found a false-negative rate as high as 8% for the detection of mediastinal metastases by PET imaging.[41,45,46]

The exact role of PET in the diagnostic algorithm of Node (N) staging has been the subject of much debate. However, staging of the mediastinum should not rely solely on PET and mediastinoscopy has still been advocated. It is suggested by some authors that one of the main values of PET is based on its high NPV for nodal disease, (estimated at > 90% in several studies).[24,25] This finding implies that eligible patients with negative mediastinal nodes on PET examinations may proceed directly to thoracotomy without the need for mediastinoscopy. False-negatives can occur in this group of patients with tumor subsequently being identified upon thoracotomy. These patients are, however, referred to by some as having “minimal N2 disease”, which confers a better prognosis.[29] De Langen and co-workers[47] have made recommendations based on the fact that the prevalence of nodal disease increases with size as seen in CT. They concluded that patients with nodes measuring < 15 mm on CT and a negative PET examination do not require mediastinoscopy, whereas those patients with negative PET but large lymph nodes on CT should nevertheless undergo invasive staging. It is suggested to avoid mediastinoscopy in patients with T1 tumors and negative PET scans.[24] The lower positive predictive value (PPV) makes cytologic or histologic confirmation necessary in case of a positive mediastinum on PET.

In patients with locally advanced but potentially operable tumors based on conventional clinical staging (stages II-IIIA), PET can detect nodal metastases that are inaccessible by cervical mediastinoscopy and that may be missed by conventional staging methods. It can change the work-up of the patient by indicating the need for a different approach to invasive lymph node sampling.[48]

## Distant Metastasis

One of the advantages of FDG-PET *vs* CT is that the whole body can be imaged. Therefore, in addition to staging the mediastinum, PET has shown promise for identifying distant metastases. There are reports of the ability of PET to detect clinically unsuspected distant metastases in 10-29% of patients.[49,50]

Bury *et al*. found PET to have an accuracy of 96% and bone scanning, 66%, in the evaluation of osseous involvement in patients with NSCLC. Although these tests were very similar in high sensitivity for bone metastasis, PET had a much higher specificity for disease than bone scan.[51] Although adrenal adenomas are readily characterized on unenhanced or enhanced CT, indeterminate adrenal nodules are common and require further evaluation by magnetic resonance imaging (MRI), biopsy or PET scanning. On PET or PET / CT imaging, the finding of FDG uptake within the adrenal gland being greater than that of the liver is a highly sensitive and specific sign of adrenal metastatic disease, with an overall diagnostic accuracy of > 92%.[52]

In the brain and genitourinary system, PET is less accurate in identifying malignancy. The high metabolic activity of the brain and the concentration and excretion of FDG in the genitourinary system make it difficult to differentiate metastatic disease from normal activity. As the brain is a common site for metastatic lung cancer, CT or MRI has been recommended.[14]

Unfortunately, it is frequently difficult to establish the diagnosis of a malignant pleural effusion because cytology of fluid obtained at thoracentesis is only positive for malignancy in 66% of patients and more invasive tests such as pleural biopsy or thoracoscopy may be required for confirmation.[24] PET has promising diagnostic accuracy for the diagnosis of pleural metastases with reported sensitivities of 92-100%, specificities of 67-71%, NPVs of 100% and PPVs of 63-79%.[53,54] Interpretation of the PET findings should take into account the results of pleural fluid analysis and the patient's recent medical history because of false positive uptake of FDG by the pleura secondary to pleural infection or inflammation after talc pleurodesis. However, a negative PET result can be useful by confirming the absence of pleural metastatic disease, particularly when the results of thoracentesis are also negative.[24]

Although PET imaging has a higher overall detection rate for metastatic disease than conventional workup, it is recommended that PET complement rather than replace conventional imaging modalities.[24]

## Treatment, Prognosis and Follow-up

Whole-body PET has potential value in treatment planning because it allows physicians to simultaneously assess for regional and metastatic disease. Therefore, PET imaging may result in the alteration of clinical staging and significantly alter management. A multivariate analysis in patients with NSCLC who were treated with either radical radiotherapy or surgery, found that the use of a cutoff of 5 for the SUV of FDG-PET in the primary tumor was the strongest prognostic factor for overall survival.[37] The use of PET clinical staging resulted in a different stage from that determined by standard methods in 62 of 102 patients (60%)-it was lowered in 20 and raised in 42.[25] In the study by Lewis *et al*., the PET findings resulted in patient management changes in 41% of the cases.[55]

Wolfgang *et al*[56] in a prospective study demonstrate that in patients with advanced NSCLC, effective chemotherapy causes a rapid reduction in the utilization of glucose by the tumor. After one cycle of platinum-based chemotherapy (21 days), a metabolic response in PET imaging was significantly correlated with the most positive response to this chemotherapy regimen. In patients without a metabolic response, the response rate was only 4%, whereas it was 71% in patients with a metabolic response. For patients with a metabolic response, the 1-year survival rate was 44%, whereas it was only 10% in patients with no metabolic response. A recent prospective study was conducted in 60 patients with stage III NSCLC who underwent neoadjuvant chemoradiotherapy before surgical resection. In these patients, a restaging PET scan 2 weeks after induction therapy was able to predict the pathological response in the primary tumor. This was later confirmed upon subsequent surgery, with a sensitivity of 86% and a specificity of 81%.[57] Another prospective study was conducted in 57 patients with locally advanced NSCLC. In these patients also who underwent restaging PET imaging after only 1 cycle of platinum-based chemotherapy, PET was able to predict the pathological response in the tumor. A fall in SUV_max_ of ≥ 20% in the primary tumor was an independent predictor of long-term survival in these patients.[58] These findings indicate that PET imaging after the initiation of chemotherapy / radiotherapy can assess the response of the primary tumor to treatment by detecting a reduction in the metabolic activity of the primary mass, which is a favorable prognostic indicator of survival.[24] Nevertheless, FDG-PET may provide a unique means to change the therapy regimen based on PET imaging findings in the early course of therapy.

**Figure 3:**
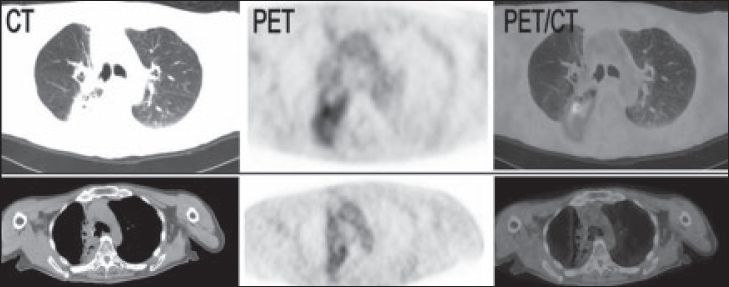
Top images: PET/CT abnormalities secondary to radiation pneumonitis. Bottom images: Resolving radiation changes 5 months later (Reprinted with permission of reference).[39]

Several studies reported that FDG-PET has a sensitivity of 98–100% and a specificity of 62–92% for the detection of recurrent malignancy after definitive treatment with surgery, chemotherapy or radiotherapy.[24,59,60] Normalization of FDG uptake after treatment appears to be a sensitive indicator of favorable response and good prognosis.[14] Hebert *et al* demonstrated that patients with negative PET scans were alive 2 years after treatment and 50% of patients with residual hypermetabolism on PET had died.[61] Sensitivity, specificity and accuracy of PET are high in differentiating recurrent malignancy from benign posttreatment changes in patients studied after therapy. Detection of recurrent disease using radiological changes is made difficult by the often extensive anatomic abnormalities that exist after definitive treatment, such as parenchymal scarring, distortion of normal bronchovascular architecture, pleural thickening and effusions and mediastinal fibrosis.[24] A PET evaluation has been shown to be more[30] useful than conventional imaging for diagnosing tumor recurrence.[12,60,62] Specificity of PET for malignant disease is lower than at initial staging because of the often coexisting inflammation secondary to radiotherapy or chemotherapy, which can appear FDG-avid on PET scans.[24] Diagnostic difficulties over the presence or absence of recurrent cancer most frequently arise after radiotherapy, in particular after three-dimensional (3D) conformal radiotherapy, which causes low-grade inflammation in the treated lung[24,63] [Figure 3]. On PET scans, areas of radiation pneumonitis characteristically appear as diffuse areas of mild to moderate FDG uptake that conform to the region of irradiated lung.[64] However, tumor recurrence is suspected where a focal site of more intense metabolic activity is seen. It is advisable to wait for a period of 3–6 months after the end of treatment before performing surveillance PET scans. Findings which arouse suspicion for tumor recurrence on a posttreatment PET scan should be confirmed by histological or cytological evidence to avoid diagnostic errors.[24]

## Radiotherapy Planning

It is the radiotherapy oncologist's goal to optimize the beneficial effects of radiation therapy while at the same time, limiting the dose to normal surrounding tissue. FDG-PET can also assist in radiation therapy planning by focusing radiation ports to precise areas of tumor activity, preventing irradiation of uninvolved areas and omission of regions of active tumor from radiation ports.[12] Accurate identification of nodal metastases is crucial for planning curative radiotherapy, particularly as routine elective nodal irradiation is no longer recommend in NSCLC.[65] Different meta-analyses have shown FDG-PET to be superior to conventional mediastinal staging using CT scans and esophageal ultrasound.[41,44] A prospective clinical trial using this approach reported isolated nodal failures in only 1 of 44 patients.[66] Vander Wal *et al* studied whether fusion PET/CT-based radiotherapy planning could improve the therapeutic ratio in 21 patients with clinical N2-N3 NSCLC. Compared with 3D CT-based planning the gross tumor volume of the nodes decreased from 13.7 to 9.9 cm^3^ on PET/CT planning (*P* = 0.011). The delivered dose could be increased from 56.0 Gy with CT to 71 Gy with PET / CT planning (*P* = 0.038), leading to better tumor control probability for a similar toxicity risk for lung, esophagus and spinal cord.[67] Due to false positive results, PET findings that can have a major impact on treatment policy should ideally be confirmed by histology.[68]

In several studies, incorporation of PET / CT imaging into treatment planning resulted in an alteration of the initial radiotherapy plan in over 50% of patients with NSCLC, compared with the use of CT alone. This was possible because of the better differentiation of the metabolically active tumor from adjacent atelectasis and by increased sensitivity for nodal metastatic disease.[24]

One group used the term ‘anatomic biologic contour’ to express the potential advantages of using PET/CT fusion radiotherapy planning. Compared with contouring treatment volumes based on CT alone, PET / CT resulted in clinically significant (> 25%) treatment volume changes in 10/19 patients and better concordance in treatment planning with different observers.[69]

Several complexities, both in fused imaging and in new radiation therapy techniques, (such as 3D conformal radiation therapy) have meant that there is a greater need for interaction between radiologists, radiation oncologists and radiation therapy physicists when planning radiotherapy for NSCLC. Radiologists should always be available for consultation when radiation treatment plans are being formulated. However, data regarding radiotherapy planning and PET/CT are still sparse and several issues remain to be solved before PET / CT is routinely used for radiotherapy planning.

### Cost-effectiveness

The general consensus is that PET can reduce needless thoracotomy rates.[29] These benefits can be quantified by calculating the incremental cost-effectiveness ratio (ICER), which may be measured in monetary terms per life year saved (LYS) or per quality adjusted life years (QALY). Gambhir *et al* used a decision analysis model to compare the cost-effectiveness of four strategies for the diagnosis and management of solitary pulmonary nodules. CT-plus-PET was the most cost-effective strategy when an intermediate pretest likelihood of 12–69% was present. In addition, a CT-plus-PET strategy over CT alone yielded cost savings of $91–2200 per patient.[70] Scott *et al* findings supported the use of thoracic PET as an adjunct to thoracic CT for preoperative staging. Furthermore, several different CT-plus-PET strategies resulted in a greater life expectancy than the CT-only strategy.[71] A recent French study used a decision tree analysis model to compare various strategies including CT only, PET for patients with negative CT and PET plus CT.[72] They concluded that employing a combination of PET and CT was the most cost-effective resulting in an ICER of -576 euros / LYS (i.e., a cost saving per life year saved). Analyses from other countries have reported similar cost savings by using PET and selective mediastinoscopy.[29,73] With PET / CT, it can be anticipated that examination costs may rise due to more expensive machinery, though this may be offset in part by improvements in staging accuracy and examination times.

## Conclusion

FDG-PET has been approved by the Health Care Finance Administration for Medicare reimbursement for diagnosing, staging and restaging lung cancer. FDG-PET and PET / CT provide a noninvasive and cost-effective strategic approach to patient selection for interventional and therapeutic procedures without contributing to increased morbidity. FDG-PET is the recommended test for evaluation of the solitary pulmonary nodule which can be as small as 7 mm, mediastinal and extrathoracic staging excluding the brain, evaluation of therapy response and restaging following treatment.[14] PET / CT has the best of both worlds of metabolic and anatomic imaging and may likely be the first choice in lung cancer imaging of the future.[14] Currently available data on PET / CT suggests that its superiority to alone PET lies principally in better T staging, but it also provides tangible benefits for N and M staging. Also PET / CT is useful in prediction of prognosis, follow-up of patients, radiotherapy planning, facilitating image-guided biopsy for definitive diagnosis as well as differentiating viable tumor from adjacent or necrotic tissue and tumor recurrence from residual scar. The clinical applications of PET / CT are still evolving and future research will determine the precise role that metabolic imaging has to play in the management of patients with lung cancer.
